# Association Between Serum Lycopene Concentrations and Diabetic Kidney Disease in the Elderly With Diabetes Mellitus: A Cross-Sectional Study From the NHANES Database

**DOI:** 10.1155/jdr/4481506

**Published:** 2025-08-08

**Authors:** Shumin Bao, Wenyi Zhu, Guojuan Zhang

**Affiliations:** ^1^Department of Nephrology, Beijing Tongren Hospital, Capital Medical University, Beijing, China; ^2^Department of Orthopaedics, Beijing Chaoyang Hospital, Capital Medical University, Beijing, China

**Keywords:** diabetic nephropathy, elderly, lycopene, NHANES database

## Abstract

This study is aimed at assessing the association between serum lycopene and its isomers with diabetic kidney disease (DKD) in order patients with diabetes mellitus (DM). Utilizing a cross-sectional design, data were extracted from the National Health and Nutrition Examination Surveys (NHANES) cycles 2003–2006 and 2017–2018. Metrics including serum lycopene, urinary albumin to creatinine ratio, and estimated glomerular filtration rate were collected. Weighted univariate and multivariate logistic regression models were conducted to explore the associations between serum lycopene isomers and DKD in order patients with DM. Subgroup analyses were conducted by different genders, complications, and medical history. A total of 960 order patients with DM were included, of whom 470 (48.96%) had DKD. After covariate adjustment, we found a *cis*-lycopene concentration range of 8.60–13.19 *μ*g/dL (Q1–Q2) was associated with lower odds of DKD (OR = 0.53, 95% CI: 0.28–0.98). This association was particularly evident among males (OR = 0.48, 95% CI: 0.25–0.93), patients with a history of hypertension (OR = 0.48, 95% CI: 0.24–0.97), patients with dyslipidemia (OR = 0.52, 95% CI: 0.27–0.99), and those without the angiotensin-converting enzyme inhibitors/angiotensin receptor blockers (ACEI/ARB) use (OR = 0.55, 95% CI; 0.31–0.99). No significant associations were observed between total lycopene, translycopene, and DKD. These findings suggest serum *cis*-lycopene may be a potential modifiable biomarker for DKD prevention in high-risk elderly populations. Concentrations may improve renal health among the elderly DM patients. Maintaining optimal *cis*-lycopene levels through dietary interventions may complement conventional renoprotective strategies. Further prospective studies are warranted to establish causal relationships and evaluate targeted lycopene supplementation regimens.

## 1. Introduction

Diabetes kidney disease (DKD), characterized by persistent albuminuria and/or reduced estimated glomerular filtration rate (eGFR), represents one of the most common prevalent complications of diabetes mellitus (DM) [1]. Globally, DKD affects approximately 20%–40% of 450 million individuals with DM [2]. Notably, elderly diabetics face disproportionate risk, and studies report > 60% DKD prevalence in those 65 years versus < 40% in younger counterparts, partly due to accelerated aged-related mitochondrial dysfunction and inflammaging [3, 4]. Without timely intervention, DKD progresses to severe complications and poor clinical outcomes. Consequently, proactive renal monitoring and targeted management strategies are critical for elderly DM patients with or at risk of DKD.

In the elderly, DKD represents not merely the manifestation of diabetic complications, but rather a complex pathological state where diabetic metabolic dysregulation and the natural aging process of the kidneys mutually exacerbate each other [5, 6]. This synergistic effect confers unique pathophysiological characteristics upon elderly diabetic nephropathy, primarily manifested as mitochondrial dysfunction [7], inflammaging [8], oxidative stress [9], and structural degeneration coupled with diminished renal regenerative capacity [10]. Lycopene, a potent lipophilic carotenoid, demonstrates well-documented anti-inflammatory, antioxidation, endothelial-protective, and antiatherosclerosis properties—physiological mechanisms of particular significance in elderly diabetic nephropathy patients [11]. Specifically, in the aging kidney, nuclear factor E2-related factor 2 (Nrf2) pathway activity is generally diminished [12]. The unique conjugated double bond structure of lycopene endows it with superior singlet oxygen quenching capability, enabling it to activate the endogenous Nrf2/ARE (antioxidant response element) antioxidant pathway by promoting Keap1 cysteine residue modification, thereby playing a critical role in the antioxidant defense system of the aging organism [13, 14]. Furthermore, lycopene can intervene in core inflammaging pathways through multiple targets, including inhibition of the NF-*κ*B signaling cascade and modulation of NOD-like receptor protein 3 inflammasome (NLRP3) inflammasome assembly [15, 16]. Several epidemiological studies have also reported associations between serum lycopene levels and the risk and prognosis of kidney disease and related conditions. A cohort study derived from the National Health and Nutrition Examination Surveys (NHANES) demonstrated that maintaining sufficient serum lycopene concentrations reduces mortality risk in chronic kidney disease (CKD) patients [17]. Another systematic review suggested a potential U-shaped association between lycopene intake and mortality risk [18]. Importantly, *cis-* and *trans-*lycopene isomers exhibit significant differences in bioavailability, antioxidant activity, anti-inflammatory effects, and signaling regulation in human and animal tissues [19].

Despite these mechanistic insights, critical knowledge gaps persist regarding the association between serum lycopene concentrations and DKD. Previous studies have not only inadequately addressed the elderly diabetic population with DKD but also relied predominantly on dietary intake estimates rather than direct serum measurements of lycopene. Therefore, our study is specifically designed to investigate the association between optimal serum lycopene concentrations and DKD in this vulnerable cohort, thereby establishing a theoretical foundation for renal health management in elderly diabetic patients.

## 2. Methods

### 2.1. Study Design and Participants

For this cross-sectional study, data were extracted from the NHANES 2003–2006 and 2017–2018 cycles, during which comprehensive measurements of lycopene isomers were systematically performed. The NHANES is a nationally representative, cross-sectional survey conducted by the National Center for Health Statistics (NCHS), a part of the Disease Control and Prevention (CDC). It is aimed at assessing the health and nutritional status of the noninstitutionalized civilian population in the United States through a combination of household interviewers, standardized physical examinations, and laboratory tests. This database uses complex, multistage, probability sampling methods based on broad population distributions. Since 1999, data have been released in biannual cycles, with approximately 5000 participants recruited per cycle. All data and documentation are publicly accessible via the official NHANES website: https://www.cdc.gov/nchs/nhanes/. The NHANES protocols were approved by the NCHS Ethics Review Board. All participants have provided informed consent.

Initially, participants aged ≥ 65 years old with diagnosed DM and available measurements of serum creatinine, urine creatinine, albumin, and serum lycopene were extracted from NHANES 2003–2006 and 2017–2018 cycles. From this cohort, we further excluded individuals with missing data on smoking status, educational levels, white blood cell (WBC), or body mass index (BMI).

### 2.2. Definition of DKD

The outcome of our study was the DKD in elderly patients with DM. DM was defined by any of the following criteria: (1) hemoglobin (HbA1c) ≥ 6.5%; (2) 2-h plasma glucose ≥ 200 mg/dL following a 75-g oral glucose tolerance test (OGTT); (3) fasting glucose ≥ 123 mg/dL; (4) self-reported physician diagnosis of DM; or (5) current use of insulin or other diabetes medication [20]. Albuminuria was defined as a urinary albumin to creatinine ratio (UACR) ≥ 30 mg/g. The eGFR was calculated using the eGFRCKD-EPI creatinine equation (mL/min/1.73m^2^) = 141 × min (Scr/*κ*, 1) *α* × max (Scr/*κ*, 1) − 1.029 × 0.993age × 1.108 (if female) × 1.159 (if black), where *κ* = 0.7 (females) or 0.9 (males); *α* = −0.329 (females) or −0.411 (males); min indicates the minimum of Scr/*κ* or 1, and max indicates the maximum of Scr/*κ* or 1 [21]. DKD was defined as the presence of persistent albuminuria (UACR ≥ 30 mg/g) and/or reduced eGFR (<60 mL/min/1.73m^2^) in individuals with diabetes.

### 2.3. Measurement of Total, *Trans*- and *Cis*-Lycopene Concentrations

Serum total, *trans*- and *cis*-lycopene concentrations were measured using a modification of high-performance liquid chromatography (HPLC) with a photodiode array detection method (https://wwwn.cdc.gov/Nchs/Nhanes/2017-2018/VITAEC_J.htm), following a rigorous validation protocol by CDC's Environmental Health Laboratory. Key validation parameters included accuracy (recovery rates 95%–105%), precision (CV < 8%), limit of detection/limit of quantitation (LOD/LOQ) (0.05/0.10 *μ*g/mL), and linearity (*R*^2^ > 0.999). All procedures adhere to Clinical Laboratory Improvement Amendment (CLIA) guidelines. The total, *trans*- and *cis*-lycopene were divided into four levels according to their quartile. For total lycopene (micrograms per deciliter): Q1: < 17.90; Q2: 17.90–27.68; Q3: 27.68–41.51; Q4: ≥ 41.51; *trans*-lycopene (micrograms per deciliter): Q1: < 9.23; Q2: 9.23–14.35; Q3: 14.35–21.70; Q4: ≥ 21.70. The serum *cis*-lycopene concentration was equal to the total lycopene minus the *trans*-lycopene (micrograms per deciliter): Q1: < 8.60; Q2: 8.60–13.19; Q3: 13.19–19.45; Q4: ≥ 19.45.

### 2.4. Potential Covariates

The potential covariates considered in this study included demographic information, physical examination, laboratory parameters, behaviors, disease, and medication history. Age, gender, and race were self-reported demographic information. Poverty to income (PIR) was calculated as the ratio of family income to the federal poverty threshold, adjusted for family size and survey year. A PIR < 1 indicates poverty status, while higher values reflect greater economic security [22]. Education level was assessed by the question “What is the highest grade or level school you have completed or the highest degree you have received?” (below high school/high school/GED or equivalent/above high school). Physical activity (PA) was expressed as the metabolic equivalent task (MET) and calculated as total weekly MET-minutes using the NHANES Physical Activity Questionnaire (PAQ) [23]. In general, the formula was as follows: Weekly MET − min = recommended MET × exercise time for corresponding activities (min/day) × the number of exercise days per week (day). Calculations were stratified by survey cycle and the specific method was exhibited in Table S1. Smoking behaviors was measured in the “smoking: cigarette use” questionnaire. In this questionnaire, participants were asked if he/she had smoked at least 100 cigarettes in their whole life, and smoked cigarettes when being questioned. If participants had smoked less than cigarettes in their life, he/she was categorized as never smoker. If participants had smoked at least 100 cigarettes in their life and still smoked when he/she answered this questionnaire, he/she was categorized as a current smoker. If the participants smoked at least 100 cigarettes, and had quit smoking now, he/she was defined as former smoker [24]. Alcohol consumption was divided into < 1 times/week, ≥ 1 times/week, and unknown, as measured by the “alcohol use” questionnaire. How much alcoholic drinks has he/she drunk in the past 12 months. The alcohol consumption included liquor, beer, wine, wine coolers, and any other types of alcoholic beverage. In the “alcohol use” questionnaire, participants were asked how much alcoholic drinks he/she had drunk in the past 12 months. The term “a drink” refers to 12 ounces of beer, 4 ounces of wine, or 1 ounce of spirits [25]. Hypertension was defined as systolic blood pressure ≥ 130 mmHg or blood pressure ≥ 80 mmHg, or self-reported disease history and taking hypertension medication [26]. Dyslipidemia was defined as total cholesterol ≥ 200 mg/dL (5.2 mmol/L) or triglyceride ≥ 150 mg/dL (1.7 mmol/L) or low-density lipoprotein cholesterol ≥ 130 mg/dL (3.4 mmol/L), high-density lipoprotein cholesterol ≤ 40 mg/dL (1.0 mmol/L), or self-reported disease history and taking lipid-lowering drugs [27]. Cardiovascular disease (CVD) was assessed by the question “Ever told had congestive heart failure/coronary heart disease/angina/heart attack?” Nephrotoxic agents use was identified based on participants' self-reported of the following drugs: antibacterial agents, antineoplastic drugs, analgesics and immunosuppressant.

### 2.5. Statistics Analysis

Continuous data were expressed as mean and standard error (SE), and the weighted *t*-test was used for comparison between groups. Categorical variables were described as the number and percentage [*N* (%)], and comparison between groups used the weighted *χ*^2^ test. Variables with a missing rate exceeding 5% were classified as the “Unknown” category, while variables with a missing rate lower than 5% were excluded. The details of the missing variables were presented in Table S2. Potential confounders were selected based on biological plausibility and prior evidence of association with DKD. Variables directly involved in defining the outcome (eGFR ad UACR for DKD diagnosis) or used to derive composite variables (height and weight for BMI calculation) were excluded to avoid overadjustment. The initial candidate confounders included age, gender, race, PIR, education level, BMI, PA, smoking status, drinking, hypertension, dyslipidemia, CVD, angiotensin converting-enzyme inhibitors/angiotensin receptor blockers (ACEI/ARB), nephrotoxic agents, WBC, and UA, which were included in a univariate logistic regression model for testing the association with DKD. Then, variables significantly associated with DKD in univariate analysis (*p* < 0.05) were included in multivariate logistic regression (Model 2) to assess the independent association between serum lycopene and DKD. The final adjusted variables in Model 1 were age, education level, PA, CVD, ACEI/ARB, WBC, and UA. Table S3 exhibited the process of covariate selection. All statistical analyses were performed using *R* v.4.20 (*R* Foundation for Statistical Computing, Vienna, Austria). Two-sided *p* value < 0.05 was considered statistically significant.

## 3. Results

### 3.1. Participant Selection and Baseline Characteristics

**Figure 1:**
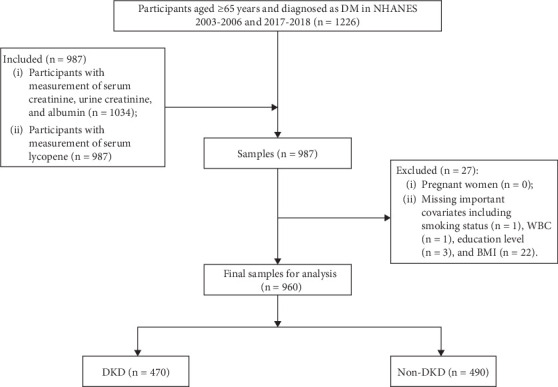
Flow diagram of eligible participant selection from NHANES 2003–2006 and 2017–2018 cycles.

Figure 1 illustrated the participant screening flowchart. In the initial pool of 987 diabetic patients aged ≥ 65 years with complete clinical data, 1 patient for missing smoking status, 1 for missing WBC, 3 for missing education level, and 22 for missing BMI were excluded. Consequently, 960 participants were finally included, with the mean age of 73.14 (0.23) years. Of whom, 470 (48.96%) had DKD.  Table 1 summarizes the baseline characteristics stratified by DKD status. Significant intergroup differences were observed in demographics, comorbidities, lifestyle, biomarkers, and lycopene isomers (all *p* < 0.05). Specifically, patients with DKD were more likely to be older (74.45 ± 0.36 vs. 72.06 ± 0.33 years), had the history of CVD (66.16% vs. 55.37%), and lower education level (below high school: 31.28% vs. 18.51%). The DKD group exhibited reduced PA (31.84% vs. 52.85%), elevated WBC (7.95 ± 0.16 vs. 7.33 ± 0.09 × 10^3^ *μ*L), and higher UA (6.30 ± 0.12 vs. 5.60 ± 0.09). While total- and *trans*-lycopene showed no significant differences in continuous or quartile analyses, *cis*-lycopene quartile demonstrated a divergent distribution (*p* = 0.043), with DKD patients overrepresented in the lowest quartile (Q1: 30.06% *vs.* 19.24%). Moreover, as expected, patients with DKD had substantially lower eGFR (62.15 ± 1.43 vs. 80.22 ± 0.75 mL/min/1.73 m^2^). No group differences were detected in gender, race, PIR, BMI, smoking status, drinking status, dyslipidemia, nephrotoxic agent use, or ACEI/ARB usage (all *p* > 0.05).

### 3.2. Associations Between Serum Lycopene Isomers With DKD

We employed two weighted logistic regression models to explore the associations between serum lycopene isomers and DKD, as depicted in Table 2. Key findings reveal the association between serum *cis*-lycopene and DKD. In crude Model 1, all lycopene isomers showed inverse associations with DKD in higher quartiles, while *cis*-lycopene exhibited the strongest effect (Q2 vs. Q1: OR = 0.50, 95% CI: 0.28–0.90, *p* = 0.022; Q4 vs. Q3: OR = 0.57, 95% CI: 0.33–0.97, *p* = 0.040). In adjusted Model 2, only *cis*-lycopene retained significance (Q2 vs. Q1: OR = 0.53, 95% CI: 0.28–0.98, *p* = 0.045). No relationships were found between concentrations of total and *trans*-lycopene and the odds of DKD (all *p* > 0.05).

### 3.3. Associations Between Serum Lycopene Isomers and DKD Stratified by Gender, Hypertension, Dyslipidemia, and ACEI/ARB

Table 3 details the stratified analyses of serum lycopene, isomers, and DKD risk across key subgroups including gender, hypertension, dyslipidemia, and ACEI/ARB. Crucially, *cis*-lycopene consistently demonstrated protective effects in clinically relevant subgroups, while other isomers showed limited robustness. In male patients, all lycopene isomers at Q2 demonstrated a significant association with DKD (OR = 0.48, 95% CI: 0.25–0.93), whereas no significant associations were observed for any isomers in female patients. Among those with comorbid hypertension, significantly reduced DKD risk was specifically observed in the Q2 *cis*-lycopene concentration quartile (OR = 0.48, 95% CI: 0.24–0.97). A similar protective association emerged for Q2 *cis*-lycopene in patients with dyslipidemia (OR = 0.52, 95% CI: 0.27–0.99). Additionally, patients without ACEI/ARB treatment exhibited significant protective effects from Q2 *cis*-lycopene concentrations (OR = 0.55, 95% CI: 0.31–0.99). No such associations were observed in ACEI/ARB-treated patients (*p* > 0.05).

## 4. Discussion

In this nationally representative study of elderly diabetic patients, we demonstrated a significant association between serum lycopene isomers and DKD, and this association varied depending on the different isomers of lycopene. Crucially, serum cis-lycopene concentrations within the range of 8.60–13.19 *μ*g/dL (corresponding to Q2) were associated with DKD (OR = 0.53, 95% CI: 0.28–0.98), particularly among males, patients with hypertension or dyslipidemia, and those not using ACEI/ARB agents. In contrast, neither total lycopene nor trans-lycopene showed significant associations with DKD after multivariable adjustment.

Age-related metabolic and renal dysfunction predispose elderly people to an increased occurrence of DM and DKD, respectively. As the prevalence of the aging population is increasing, the prevalence of DKD in the elderly is likely to increase. Several previous studies have shown the role of inflammation and oxidative stress in the pathogenesis of DKD [4, 28]. Excessive oxidative stress leads directly to the damage of the renal interstitium, glomerulus, and podocytes, which in turn impairs renal function. Since oxidative stress is also closely associated with inflammatory cells, the two often coexist and activate each other. Reactive oxygen species (ROS) can mediate renal inflammation and accelerate the development of DKD. In addition, high glucose also affects renal function by affecting renal blood flow [29].

Lycopene, a member of the carotenoid family, has powerful properties to alleviate oxidative stress and suppress inflammation, and it is far more effective at scavenging free radicals than other carotenoids and vitamin E. Absorbed from food, lycopene is mainly distributed in adipose tissue, liver, and serum [30]. Several studies have focused on the association between serum lycopene and kidney diseases. A cohort study from Zhong et al. [17] reported that higher serum lycopene concentrations were independently related to a decreased risk of all-cause and CVD mortality in CKD patients, and for every 1% increase in serum concentration, all0cause mortality decreased by 0.7%. This study suggested that appropriate lycopene concentration has potential benefits for the prognosis of CKD patients. Similar results were also reported by Hu et al. [31] that high serum lycopene concentrations reduced all-cause mortality by 15% among CKD patients. Zhang et al. [19] explored the association between different isomers of lycopene and all-cause and CVD mortality, and the results showed that *cis*-lycopene had a U-shaped relationship with mortality, while *trans*-lycopene had an inverse relationship with it. Our study found that the lower odds of DKD among the elderly DM patients were only associated with *cis*-lycopene at Q1-Q2 concentration after adjusting for all covariates. However, no statistical association was observed between serum total lycopene and *trans*-lycopene concentrations with the odds of DKD in our study population. *Cis*- and *trans*-lycopene were two isomers commonly found in human and animal tissues, and the human body contains mainly the *cis*-isomer [32]. Several study groups have proposed that *cis*-lycopene is more readily absorbed than the all-trans lycopene isomer due to the shorter length of *cis*-lycopene, the greater solubility of *cis*-isomers in mixed micelles, and/or as a result of the lower tendency of the *cis*-lycopene isomer to aggregate [33, 34]. Since *cis*-lycopene has better bioavailability, it can inhibit ROS production in renal tubular epithelial cells and reduce mitochondrial damage to improve kidney health. The renal protective effects of *cis*-lycopene may be mediated through its dual modulation of key redox pathways. The *cis*-lycopene inhibits NADPH oxidase (NOX) complex assembly, particularly the NOX4 isoform, which is abundantly expressed in renal tubular cells [35]. This reduces superoxide anion generation at the mitochondrial level. Secondly, by dissociating Nrf2 from Keap1, *cis*-lycopene facilitates Nrf2 translocation, upregulating antioxidant enzymes (heme oxygenase 1 [HO-1], NAD(P)H:quinone oxidoreductase 1 [NQO1], and superoxide dismutase [SOD]) that neutralize ROS in glomerular podocytes [36]. Concomitant suppression of NF-*κ*B signaling attenuates ROS-induced proinflammatory cytokine production (tumor necrosis factor-*α* [TNF-*α*] and interleukin-6 [IL-6]) in renal mesangial cells [14]. This multitarget action mitigates oxidative damage to renal lipids, DNA, and proteins—hallmarks of DKD progression.

We only observed the association between serum lycopene concentration and DKD at Q2 concentration level, and no such association was observed at lower or higher concentrations. We speculate that this nonlinear phenomenon may be related to the following three physiological mechanisms. First, at higher concentrations, saturation of lycopene transporters such as SR-B1 and CD36 may limit cellular uptake in renal tissues [37]. Second, excessive *cis*-lycopene may paradoxically exhibit pro-oxidant activity under high oxidative stress conditions via auto-oxidation, as demonstrated in hyperglycemic renal models [38]. Third, elevated *cis*-isomer levels could inhibit enzymatic conversion to bioactive metabolites such as apo-10⁣′-lycopemoids, reducing nephroprotective efficacy [39]. This nonlinear association remains robust within subgroups, especially among males with a history of hypertension and dyslipidemia, and without ACEI/ARB treatment. Although there are significant sex differences in the incidence and clinical manifestations of DKD, sex-based differences in the clinical aspects of DKD have not been fully explored [40]. Male gender appears to be an independent risk factor for DKD, especially when the albuminuria phenotype is considered. But females are at higher risk for advanced renal insufficiency and common DKD risk factors, and these differences are most pronounced in older subjects [41]. Allore et al. [42] suggested that lycopene concentrations were significantly higher in females than in males. Further large-scale and gender-balanced cohort studies are needed to evaluate the association between serum lycopene and DKD among elderly DM patients. Hypertension and dyslipidemia are common comorbidities of DKD, which can accelerate the progression of DKD [43]. ACEI/ARBs have been recommended by Kidney Disease: Improving Global Outcomes for controlling blood pressure, reducing proteinuria, and delaying the deterioration of renal function. While this observational evidence cannot establish causality, maintaining serum *cis*-lycopene within this range may represent a biomarker of renal health in elderly diabetics with hypertension, dyslipidemia, or nonuse of ACEI/ARB. Clinical applications would require verification through interventional trials.

Herein, we provided scientific evidence regarding the association between serum lycopene isomers and renal health in elderly diabetic patients. Our findings suggest that sustaining serum cis-lycopene concentrations within the optimal range (8.60–13.19 *μ*g/dL) may be associated with better renal outcomes in this population. Given that lycopene cannot be endogenously synthesized, achieving adequate levels through dietary sources appears crucial. Although no official daily intake recommendation exists, thermal processing of tomato-based foods (accounting for ~85% of dietary lycopene) enhances *cis*-isomer bioavailability via micellar solubilization [44]. These observations should be interpreted considering several limitations. First and foremost, as a cross-sectional study, our analysis is inherently limited in establishing causal relationships between serum lycopene and DKD. The observed associations may reflect reverse causality, such as DKD progression altering lycopene metabolism, or residual confounding from unmeasured factors. While we rigorously adjusted for key clinical and socioeconomic confounders, prospective cohort studies or randomized trials are needed to verify whether lycopene supplementation can prevent DKD onset or progression. Second, although we adjusted for as many potential confounders as possible, our study remains susceptible to residual confounding due to unmeasured variables. Serum lycopene levels are significantly influenced by dietary intake of lycopene-rick foods. However, data from the NHANES did not comprehensively assess broader dietary patterns, such as overall fruit and vegetable consumption, fat intake, and processed food intake—which may independently influence the risk of DKD. Moreover, NHANES lacks detailed assessments of unrecorded nonsteroidal anti-inflammatory drug (NSAID)—these data gaps could introduce bias into our assessment of the exposure-outcome association. Failure to account for lycopene supplementation, particularly if high-risk individuals engage in self-medication, may attenuate the observed association. Finally, the generalizability of our findings may be limited by external validity concerns. NHANES data represent the US noninstitutionalized civilian population; thus, extrapolating results to populations with distinct genetic backgrounds, environmental exposures, lifestyles, dietary habits, or healthcare systems requires caution.

To validate and extend our findings, the following targeted investigations are warranted: first, prospective cohort studies with repeated measurements of serum lycopene and standardized assessments of dietary patterns such as validated food frequency questionnaires to minimize residual confounding and establish temporal relationships; second, interventional trials examining the effects of lycopene supplementation on DKD progression in high-risk diabetic populations, particularly focusing on renal endpoints; third, mechanistic studies to elucidate the biological pathways linking lycopene to DKD pathogenesis, including its effects on oxidative stress, inflammation, and podocyte dysfunction in experimental models; fourth, studies incorporating objective biomarkers to better control for unmeasured confounders such as undocumented drug use.

## 5. Conclusion

In this study, serum *cis*-lycopene concentration within the range of 8.60–13.19 *μ*g/dL (corresponding to the second quartile, Q2) was inversely associated with DKD among elderly diabetic patients. No relationship was found between serum total lycopene and *trans*-lycopene with DKD in our study population. These findings suggest that maintaining optimal serum *cis*-lycopene concentration within this specific range may confer renal protective benefits in the geriatric diabetes populations.

## Figures and Tables

**Table 1:** Baseline characteristics of DM patients according to with or without DKD.

**Variables**	**Total (** **n** = 960**)**	**Non-DKD (** **n** = 490**)**	**DKD (** **n** = 470**)**	**Statistics**	**p**
Age, years, mean (± SE)	73.14 (± 0.23)	72.06 (± 0.33)	74.45 (± 0.36)	*t* = 4.879	< 0.001
Gender, *n* (%)				*χ* ^2^ = 3.928	0.054
Female	439 (50.67)	250 (54.64)	189 (45.88)		
Male	521 (49.33)	240 (45.36)	281 (54.12)		
Race, *n* (%)				*χ* ^2^ = 0.323	0.744
Non-Hispanic White	466 (74.39)	221 (75.34)	245 (73.24)		
Non-Hispanic Black	206 (10.96)	107 (10.34)	99 (11.70)		
Mexican American	162 (4.99)	94 (5.23)	68 (4.69)		
Other race	126 (9.67)	68 (9.09)	58 (10.36)		
PIR, *n* (%)				*χ* ^2^ = 0.422	0.655
< 1	140 (8.04)	70 (7.23)	70 (9.02)		
≥ 1	730 (84.15)	369 (84.66)	361 (83.53)		
Unknown	90 (7.81)	51 (8.11)	39 (7.46)		
Education level, *n* (%)				*χ* ^2^ = 6.954	0.002
Below high school	351 (24.30)	160 (18.51)	191 (31.28)		
High school/GED or equivalent	249 (29.84)	135 (30.88)	114 (28.59)		
Above high school	360 (45.86)	195 (50.61)	165 (40.13)		
Height (cm), mean (± SE)	165.55 (± 0.34)	165.28 (± 0.60)	165.87 (± 0.56)	*t* = 0.619	0.539
Weight (kg), mean (± SE)	85.38 (± 0.87)	84.92 (± 1.13)	85.94 (± 1.32)	*t* = 0.593	0.556
BMI (kg/m^2^), *n* (%)				*χ* ^2^ = 0.071	0.925
< 25	169 (14.87)	82 (14.30)	87 (15.57)		
25–30	336 (32.90)	176 (33.11)	160 (32.65)		
≥ 30	455 (52.22)	232 (52.59)	223 (51.78)		
PA, MET·(min/week), *n* (%)				*χ* ^2^ = 9.269	<0.001
< 450	231 (23.66)	110 (19.84)	121 (28.27)		
< 750	54 (5.81)	28 (5.53)	26 (6.14)		
≥ 750	372 (43.32)	227 (52.85)	145 (31.84)		
Unknown	303 (27.21)	125 (21.78)	178 (33.75)		
Smoking, *n* (%)				*χ* ^2^ = 1.423	0.247
Never	454 (48.33)	243 (51.11)	211 (44.97)		
Former	430 (44.28)	206 (41.55)	224 (47.56)		
Current	76 (7.39)	41 (7.33)	35 (7.47)		
Drinking, times/week, *n* (%)				*χ* ^2^ = 1.644	0.199
< 1	400 (43.94)	213 (45.47)	187 (42.10)		
≥ 1	139 (16.48)	74 (18.29)	65 (14.30)		
Unknown	421 (39.58)	203 (36.24)	218 (43.60)		
Hypertension, *n* (%)				*χ* ^2^ = 4.074	0.050
No	84 (8.07)	50 (10.15)	34 (5.56)		
Yes	876 (91.93)	440 (89.85)	436 (94.44)		
Dyslipidemia, *n* (%)				*χ* ^2^ = 1.378	0.247
No	94 (7.35)	56 (8.46)	38 (6.01)		
Yes	866 (92.65)	434 (91.54)	432 (93.99)		
CVD, *n* (%)				*χ* ^2^ = 6.203	0.017
No	389 (39.73)	232 (44.63)	157 (33.84)		
Yes	571 (60.27)	258 (55.37)	313 (66.16)		
ACEI/ARB, *n* (%)				*χ* ^2^ = 8.345	0.006
No	620 (65.25)	331 (71.07)	289 (58.23)		
Yes	340 (34.75)	159 (28.93)	181 (41.77)		
Nephrotoxic agents, *n* (%)				*χ* ^2^ = 0.108	0.744
No	721 (76.57)	370 (75.99)	351 (77.28)		
Yes	239 (23.43)	120 (24.01)	119 (22.72)		
WBC, 10^3^/uL, mean (± SE)	7.61 (± 0.09)	7.33 (± 0.09)	7.95 (± 0.16)	*t* = 3.245	0.002
UA (mg/dL), ,mean (± SE)	5.92 (± 0.08)	5.60 (± 0.09)	6.30 (± 0.12)	*t* = 5.133	< 0.001
EGFR (mL/min/1.73 m^2^), mean (± SE)	72.02 (± 0.82)	80.22 (± 0.75)	62.15 (± 1.43)	*T* = −10.558	< 0.001
UACR (mg/g), mean (± SE)	101.79 (± 15.74)	11.28 (± 0.54)	210.85 (± 35.58)	*t* = 5.628	< 0.001
Total lycopene (*μ*g/dL), mean (± SE)	31.83 (± 1.19)	33.13 (± 1.77)	30.26 (± 1.03)	*t* = −1.579	0.122
Total lycopene (*μ*g/dL), *n* (%)				*χ* ^2^ = 1.884	0.144
Q1	238 (23.13)	105 (18.97)	133 (28.14)		
Q2	240 (25.78)	131 (27.14)	109 (24.14)		
Q3	239 (24.52)	129 (25.59)	110 (23.23)		
Q4	243 (26.57)	125 (28.30)	118 (24.48)		
*Trans*-lycopene (*μ*g/dL), mean (± SE)	16.85 (± 0.69)	17.61 (± 1.03)	15.93 (± 0.58)	*t* = −1.605	0.116
*Trans*-lycopene (*μ*g/dL), n (%)				*χ* ^2^ = 2.052	0.112
Q1	238 (24.37)	106 (20.47)	132 (29.06)		
Q2	239 (24.62)	136 (27.36)	103 (21.31)		
Q3	240 (24.46)	122 (23.89)	118 (25.16)		
Q4	243 (26.55)	126 (28.28)	117 (24.47)		
*Cis*-lycopene (*μ*g/dL), mean (± SE)	14.99 (± 0.51)	15.52 (± 0.75)	14.34 (± 0.49)	*t* = −1.474	0.148
*Cis*-lycopene (*μ*g/dL), *n* (%)				*χ* ^2^ = 2.879	0.043
Q1	237 (24.15)	105 (19.24)	132 (30.06)		
Q2	242 (26.06)	137 (28.94)	105 (22.59)		
Q3	237 (23.39)	121 (23.98)	116 (22.67)		
Q4	244 (26.40)	127 (27.84)	117 (24.68)		

*Note: t*: weighted *t* test; *χ*^2^: Rao–Scott chi-square test.

Abbreviations: ACEI, angiotensin-converting enzyme inhibitors; ARB, angiotensin receptor blockers; BMI, body mass index; CVD, cardiovascular disease; DKD, diabetes kidney disease; DM, diabetes mellitus; eGFR, estimated glomerular filtration rate; met, metabolic equivalent task; PA, physical activity; PIR, income-to-poverty ratio; Q1, Quartile 1; Q2, Quartile 2; Q3, Quartile 3; Q4, Quartile 4; SE, standard error; UA, uric acid; UACR, urinary albumin to creatinine ratio; WBC, white blood cell.

**Table 2:** Association between serum lycopene and its isomers with DKD.

**Variables**	**Model 1**		**Model 2**	
	**OR (95% CI)**	**p**	**OR (95% CI)**	**p**
Total lycopene				
Q1	Ref		Ref	
Q2	0.60 (0.32–1.12)	0.105	0.68 (0.36–1.28)	0.223
Q3	0.61 (0.38–0.98)	0.040	0.77 (0.49–1.20)	0.236
Q4	0.58 (0.35–0.98)	0.041	0.88 (0.50–1.55)	0.645
*Trans*-lycopene				
Q1	Ref		Ref	
Q2	0.55 (0.32–0.93)	0.028	0.67 (0.39–1.16)	0.148
Q3	0.74 (0.42–1.33)	0.306	0.95 (0.54–1.65)	0.848
Q4	0.61 (0.37–1.01)	0.053	0.96 (0.55–1.68)	0.879
*Cis*-lycopene				
Q1	Ref		Ref	
Q2	0.50 (0.28–0.90)	0.022	0.53 (0.28–0.98)	0.045
Q3	0.60 (0.38–0.96)	0.034	0.83 (0.52–1.32)	0.417
Q4	0.57 (0.33–0.97)	0.040	0.74 (0.39–1.38)	0.331

*Note:* Model 1: crude model; Model 2: adjustment for age, education, PA, CVD, ACEI/ARB, WBC, and UA.

Abbreviations: CI, confidence intervals; DKD, diabetic kidney disease; DM, diabetes mellitus; OR, odds ratio; Q1, Quartile 1; Q2, Quartile 2; Q3, Quartile 3; Q4, Quartile 4; Ref, reference.

**Table 3:** Association between serum lycopene isomers and DKD based on different gender, complications, and medication treatment.

**Subgroups (outcome/total)**	**Total lycopene**		***Trans*-lycopene**		***Cis*-lycopene**	
	**OR (95% CI)**	**p**	**OR (95% CI)**	**p**	**OR (95% CI)**	**p**
Male (*n* = 281/521)						
Q1	Ref		Ref		Ref	
Q2	0.43 (0.21–0.90)	0.027	0.43 (0.19–0.99)	0.047	0.48 (0.25–0.93)	0.032
Q3	0.87 (0.43–1.80)	0.705	1.05 (0.49–2.24)	0.900	1.02 (0.55–1.91)	0.938
Q4	1.06 (0.56–2.02)	0.854	1.08 (0.56–2.08)	0.805	0.95 (0.44–2.03)	0.892
Female (*n* = 189/439)						
Q1	Ref		Ref		Ref	
Q2	0.92 (0.38–2.23)	0.842	0.90 (0.42–1.94)	0.782	0.53 (0.22–1.28)	0.150
Q3	0.68 (0.35–1.32)	0.242	0.87 (0.43–1.75)	0.685	0.66 (0.32–1.36)	0.253
Q4	0.56 (0.24–1.33)	0.182	0.67 (0.30–1.48)	0.307	0.48 (0.20–1.13)	0.091
Nonhypertension (*n* = 34/84)						
Q1	Ref		Ref		Ref	
Q2	2.35 (0.01–406.12)	0.549	1.85 (0.01–337.49)	0.661	2.66 (0.03–219.22)	0.440
Q3	3.27 (0.01–791.87)	0.451	5.17 (0.03–898.31)	0.304	3.62 (0.02–702.75)	0.404
Q4	16.31 (0.23–1176.74)	0.107	14.47 (0.18–1154.10)	0.120	16.80 (0.29–967.31)	0.096
Hypertension (*n* = 436/876)						
Q1	Ref		Ref		Ref	
Q2	0.62 (0.30–1.27)	0.183	0.63 (0.33–1.18)	0.144	0.48 (0.24–0.97)	0.042
Q3	0.69 (0.42–1.13)	0.137	0.86 (0.48–1.55)	0.611	0.75 (0.44–1.28)	0.283
Q4	0.78 (0.41–1.48)	0.432	0.86 (0.45–1.64)	0.635	0.65 (0.32–1.30)	0.215
Nondyslipidemia (*n* = 38/94)						
Q1	Ref		Ref		Ref	
Q2	1.01 (0.21–4.94)	0.991	1.26 (0.22–7.30)	0.776	0.89 (0.11–7.00)	0.903
Q3	1.04 (0.12–9.28)	0.970	0.93 (0.17–5.21)	0.926	2.61 (0.31–21.80)	0.344
Q4	2.44 (0.30–19.95)	0.372	1.74 (0.21–14.09)	0.575	3.78 (0.30–47.15)	0.273
Dyslipidemia (*n* = 432/866)						
Q1	Ref		Ref		Ref	
Q2	0.68 (0.34–1.34)	0.255	0.65 (0.35–1.18)	0.151	0.52 (0.27–0.99)	0.048
Q3	0.75 (0.46–1.24)	0.252	0.94 (0.53–1.66)	0.816	0.79 (0.48–1.31)	0.349
Q4	0.83 (0.48–1.43)	0.494	0.91 (0.52–1.60)	0.749	0.68 (0.37–1.27)	0.215
Non-ACEI/ARBEI treatment (*n* = 289/620)						
Q1	Ref		Ref		Ref	
Q2	0.70 (0.39–1.25)	0.218	0.84 (0.43–1.61)	0.580	0.55 (0.31–0.99)	0.045
Q3	0.89 (0.57–1.38)	0.581	1.25 (0.67–2.35)	0.476	0.94 (0.57–1.57)	0.811
Q4	1.01 (0.52–1.97)	0.968	1.22 (0.64–2.32)	0.538	0.93 (0.47–1.83)	0.835
ACEI/ARBEI treatment (*n* = 181/340)						
Q1	Ref		Ref		Ref	
Q2	0.61 (0.22–1.71)	0.334	0.44 (0.16–1.18)	0.099	0.48 (0.19–1.22)	0.118
Q3	0.58 (0.25–1.31)	0.182	0.53 (0.22–1.27)	0.150	0.68 (0.34–1.36)	0.263
Q4	0.84 (0.34–2.03)	0.684	0.75 (0.30–1.83)	0.508	0.58 (0.21–1.59)	0.282

*Note:* Models: adjustment for age, education, PA, CVD, ACEI/ARB, WBC, and UA.

Abbreviations: CI, confidence intervals; DKD, diabetic kidney disease; DM, diabetes mellitus; OR, odds ratio; Q1, Quartile 1; Q2, Quartile 2; Q3, Quartile 3; Q4, Quartile 4; Ref, reference.

## Data Availability

The data that support the findings of this study are openly available in the NHANES database at https://wwwn.cdc.gov/nchs/nhanes/.

## Supporting Information

Supporting Information Additional supporting information can be found online in the Supporting Information section. The supporting information includes the detailed descriptions and methods for the calculation of physical activity, the handling of missing values, and the screening of covariates used in this study. These supporting tables provide comprehensive information necessary to replicate the analyses conducted in this study. Table S1: The specific calculation method for physical activity. Table S2: Details and handling methods of missing value. Table S3: The results of univariable logistic regression analysis.
