# Supplementary material for: Association Between Serum Lycopene Concentrations and Diabetic Kidney Disease in the Elderly With Diabetes Mellitus: A Cross-Sectional Study From the NHANES Database
Source: J Diabetes Res. 2025 Aug 8;2025:4481506. doi: 10.1155/jdr/4481506 (PMC12356673; doi:10.1155/jdr/4481506)
Supplement: Supporting Information — Additional supporting information can be found online in the Supporting Information section. The supporting information includes the detailed descriptions and methods for the calculation of physical activity, the handling of missing values, and the screening of covariates used in this study. These supporting tables provide comprehensive information necessary to replicate the analyses conducted in this study. Table S1: The specific calculation method for physical activity. Table S2: Details and handling methods of missing value. Table S3: The results of univariable logistic regression analysis. [file 4481506.f1.docx]

Supplementary Table 1 The specific calculation method for PA

| **Component** | **NHANES 2003-2006 Cycle** | **NHANES 2017-2018 Cycle** |
| --- | --- | --- |
| Core formula | PA= PADMETS $\times$ PADDURAT $\times(\frac{PADTIMES}{4})$ | PA = PAQ655 $\times$PAD660 $\times$8 + PAQ640 $\times$PAD645 $\times$4 + PAQ670 $\times$PAD675 $\times$4 + PAQ610 $\times$PAD615$\times8$ + PAQ625 $\times P$AD630 $\times$4 |
| Frequency variables | PADTIMES | PAQ655, PAQ670, PAQ610, PAQ640 |
| Time of duration | PADDURA | PAD660, PAD645, PAD675, PAD615, PAD630 |
| Key transformation | PADTIMES (convert the 30-day frequency to a weekly frequency) | None (original variable is already in weekly frequency) |
| Missing data handle | Unknown | If the answer to the following question is "No", then the activity = 0:  PAQ605, PAQ620, PAQ635, PAQ650, PAQ665 |

Note, NHANES, National Health and Nutrition Examination Surveys; PA, physical activity (unit: MET·min/week).

PADTIMES, *#*of times did activity in past 30 days; PADDURA, average duration of activity (minutes); PAD655, PAD660, Minutes vigorous recreational activities; PPAD645, minutes walk/bicycle for transportation; PAD675, minutes moderate recreational activities; PAD615, minutes vigorous-intensity work; PAD630, minutes moderate-intensity work; AQ605, Vigorous work activity; PAQ620, Moderate work activity; PAQ635, Walk or bicycle; PAQ650, Vigorous recreational activities; PAQ665, Moderate recreational activities; PAQ655, Days vigorous recreational activities; PAQ670, Days moderate recreational activities; PAQ610, Days vigorous work; PAQ640, Number of days walk or bicycle;

Original data source in NHANES, for 2003-2006 cycles, <https://wwwn.cdc.gov/Nchs/Nhanes/2003-2004/PAXRAW_C.htm>; for 2017-2018 cycle, <https://wwwn.cdc.gov/Nchs/Nhanes/2017-2018/PAQ_J.htm>.

Supplementary Table 2 Details and handling methods of missing value

| Variables | Total samples | Number of missing values | Rate of missing | Total samples | Handling method |
| --- | --- | --- | --- | --- | --- |
| Smoking | 987 | 1 | 0.1% | 986 | Delete |
| WBC | 987 | 1 | 0.1% | 986 | Delete |
| Education | 987 | 3 | 0.3% | 984 | Delete |
| Weight | 987 | 15 | 1.52% | 972 | Delete |
| Height | 987 | 20 | 2.03% | 967 | Delete |
| BMI | 987 | 22 | 2.23% | 965 | Delete |
| PIR | 987 | 93 | 9.42% | 894 | Unknown |
| PA | 987 | 316 | 32.02% | 671 | Unknown |
| Drinking | 987 | 439 | 44.48% | 548 | Unknown |

Note, WBC, white blood cell; BMI, body mass index; PIR, poverty-to-income ratio; PA, physical activity.

Supplementary Table 3 The results of univariable logistic regression analysis

| Variables | OR (95% CI) | *P* |
| --- | --- | --- |
| Age | 1.08 (1.04-1.11) | <0.001 |
| Gender |  |  |
| Female | Ref |  |
| Male | 1.42 (0.99-2.03) | 0.054 |
| Race |  |  |
| Non-Hispanic White | Ref |  |
| Non-Hispanic Black | 1.16 (0.76-1.78) | 0.477 |
| Mexican American | 0.92 (0.62-1.38) | 0.687 |
| Other Race | 1.17 (0.63-2.18) | 0.608 |
| PIR |  |  |
| <1 | Ref |  |
| ≥1 | 0.79 (0.44-1.41) | 0.417 |
| Unknown | 0.74 (0.34-1.59) | 0.427 |
| Education |  |  |
| Below High School | Ref |  |
| High School/GED or Equivale | 0.55 (0.36-0.84) | 0.007 |
| Above High School | 0.47 (0.31-0.71) | 0.001 |
| BMI |  |  |
| <25 | Ref |  |
| 25~30 | 0.91 (0.47-1.74) | 0.760 |
| ≥30 | 0.90 (0.50-1.63) | 0.731 |
| PA |  |  |
| <450 | Ref |  |
| <750 | 0.78 (0.38-1.58) | 0.480 |
| ≥750 | 0.42 (0.26-0.69) | 0.001 |
| Unknown | 1.09 (0.72-1.64) | 0.682 |
| Smoking |  |  |
| No | Ref |  |
| Former | 1.30 (0.96-1.77) | 0.092 |
| Now | 1.16 (0.64-2.10) | 0.620 |
| Drinking |  |  |
| <1 | Ref |  |
| ≥1 | 0.84 (0.50-1.42) | 0.513 |
| Unknown | 1.30 (0.89-1.91) | 0.176 |
| Hypertension |  |  |
| No | Ref |  |
| Yes | 1.92 (0.99-3.71) | 0.053 |
| Dyslipidemia |  |  |
| No | Ref |  |
| Yes | 1.44 (0.77-2.72) | 0.249 |
| CVD |  |  |
| No | Ref |  |
| Yes | 1.58 (1.09-2.28) | 0.017 |
| ACEI/ARBEI |  |  |
| No | Ref |  |
| Yes | 1.76 (1.18-2.62) | 0.006 |
| Nephrotoxic Agents |  |  |
| No | Ref |  |
| Yes | 0.93 (0.60-1.45) | 0.744 |
| WBC | 1.13 (1.04-1.23) | 0.004 |
| UA | 1.40 (1.23-1.59) | <0.001 |

Note, OR, odds ratio; CI, confidence interval; Ref, reference; PIR, poverty-to-income ratio; GED, general equivalent diploma; BMI, body mass index; PA, physical activity; CVD, cardiovascular disease; ACEI: angiotensin converting enzyme inhibitors; ARB: angiotensin receptor blockers; WBC, white blood cell; UA, uric acid.
